# Supplementary material for: Fragmentation and inefficiencies in US equity markets: Evidence from the Dow 30
Source: PLoS One. 2020 Jan 22;15(1):e0226968. doi: 10.1371/journal.pone.0226968 (PMC6975550; doi:10.1371/journal.pone.0226968)
Supplement: S1 Appendix — Describes several classes of market participants and how they interact. (PDF) [file pone.0226968.s001.pdf]

1 Market Participants

1.1 Traders

The four broad classes of traders have different market objectives, and thus generally have different mechanisms for interacting with the market. Retail investors generally have a small amount of capital and thus interact with the market indirectly, usually through their broker. Since their orders are so small in relation to both the value of total market transactions and the size of the inventory of the executing broker-dealer, their orders are often *internalized* (*i.e.* matched against the inventory of their broker-dealer rather than finding a counter-party in the lit market) [1]. For example, if investor A wishes to sell 100 shares of AAPL at the market price to sell, it is likely that, within a large brokerage, investor B wishes to buy 100 shares of AAPL at the market price to buy; these orders can then be executed at the midpoint of the prices. Alternatively, the broker-dealer may choose to be the counter-party to both traders, using its own inventory of equities and capital.

Institutional investors represent an institution, such as a large corporation, university, or state pension fund. They are typically far more highly capitalized than a retail investor, and thus their orders are likely to interact more directly (whether through their brokerage or with a market maker) with the NMS.

Brokerages execute orders on behalf of their clients. They may do this by contacting market-makers, who will execute trades on behalf of the brokerage, or they may themselves be a market-maker or broker-dealer. Brokerages may enter into contracts with market-makers, who agree to buy some percentage of the brokerage's order flow [2]. Securities and Exchange Commission (SEC) (the chief regulatory body of equity markets in the US) regulation requires brokers to guarantee their clients the "best execution" for their trades, which may include most competitive price for their trades (i.e., highest possible bid price and lowest possible offer price) [3]. As has been previously identified elsewhere [4, 5], this regulatory requirement for "Best Price" execution fails to consider implications from special relativity [6]; namely, that it is impossible to determine whether two distinct events occur at the same time if those events are geographically separated in space.

Market-makers are responsible for ensuring the market's liquidity. They quote a buy and sell price for a set of traded assets at all times, and stand ready to buy or sell an amount of those assets at their respective prices [7]. Exchanges can establish designated market-makers, who are responsible for"making the market" in a specific asset [8].

1.2 Market centers

Exchanges in the NMS are privately-owned venues on which securities are traded. They are extensively regulated by the SEC and are required by law to provide the best possible execution price (under most circumstances) to their customers [3, 9]. For each equity, each exchange maintains a local order book that aggregates the orders submitted by market participants. These local order books contain information about resting limit orders, updated by order flow, including the side (buy/sell), limit price, size, and execution modifiers that give the market participant greater control over how and when their order is executed. Using their local book and proprietary matching software, exchanges match buyers with sellers.

In 2016 there were 13 major stock exchanges:

a) NYSE (3): main exchange; ARCA, primarily for trading exchange-traded funds (ETFs); and MKT, the smallest of the NYSE family

b) NASDAQ (3): main exchange; BX, the Boston stock exchange; and PSX, the Philadelphia stock exchange

c) BATS (4): BATS and BATS Y; EDGX and EDGA. These exchanges are now owned by CBOE, effective early 2017.

d) IEX: the Investors Exchange, which was a ATS until 17 June 2016

e) CHX: the Chicago stock exchange

f) NSX: the National Stock Exchange and by far the smallest stock exchange in terms of shares traded. It has a long history of trading intermittently, with pauses in operation of duration longer than a year.

Though each exchange keeps offices in its namesake city, trading actually occurs (via each exchange's matching engine) in one of three data centers in northern New Jersey [10].

ATSs, colloquially known as "dark pools", are market centers on which invited participants may trade equity and other securities. While regulated by the SEC, ATSs are not required to publish quotes and are subject to less scrutiny than are the exchanges. ATSs are not required to publish the location of their matching engine(s), and as a rule their location is generally not known to the public. Public SEC filings contain a location for each registered ATS, though it may simply be an office and not the location of the matching engine.

1.3 Regulatory mechanisms

The National Market System is regulated primarily by the SEC. The equities industry also self-regulates through the Financial Industry Regulatory Authority (FINRA), which charges itself with regulating member brokerages and exchanges. While an authoritative institution, it does not have law enforcement power itself and must refer suspected violations of securities law to the SEC for enforcement. (FINRA has some ability to provide incentives and penalties to member organizations, such as expulsion.) The Securities Information Processor (SIP), mandated by SEC regulation, is a digital information processor on which all quotes, trades, and administrative messages such as trading halts and limit-up / limit-down (LULD) messages are recorded and through which information can be disseminated to exchanges, ATSs, and other market participants. The SIP constructs the NBBO from this data, which forms the basis of the notion of "best price" for the National Market system. There are three SIP data collection "tapes", two of which (A and B) are located at the NYSE data center in Mahwah, NJ, and one of which (C) is located at the NASDAQ data center in Carteret, NJ.

In addition to the SIP tapes mentioned above, there are two FINRA-operated Trade Reporting Facilities (TRFs), one each in Mahwah and Carteret. ATSs are required to report trades to the TRFs, which in turn report the trades to the correct SIP tape [11, 12].

Other regulatory machinery exists to prevent the "overheating" of markets in the form of price changes deemed excessive [13]. There are two types of these mechanisms: individual-stock limit-up, limit-down (LULD) mechanisms and market-wide circuit-breakers. Individual stock LULD mechanisms set price bands of 5%, 10%, and 20% for each individual stock based on prices in the immediate trailing five-minute trading period. If the stock's price exits the bands and does not return within a fifteen second time period, a five-minute trading halt for that stock is initiated. Similarly, market-wide circuit breakers (set at 7%, 13%, and 20%) initiate halts in trading if the S&P 500 breaches these bands. A breach of the first two levels results in a market-wide trading halt for 15 minutes, while a breach of the last band results in a trading halt for the rest of the trading day.

Regulatory influence on the market is not limited to price reporting and circuit-breaker mechanisms. Beginning in 2016, the SEC instituted a live-market experiment in which some securities would be quoted in minimum increments greater than a penny (which is the current minimum increment at which prices are quoted for all stocks with a share price greater than $1.00) [14]. Known as the tick-size pilot program (or tick pilot), this program directly alters the pricing mechanism and fundamental price quantization and thus may have an effect on market dynamics.

References

1. Securities US, Commission E. Trade Execution; 2013.
2. Securities US, Commission E. Payment for Order Flow; 2007.
3. Securities US, Commission E. Best Execution; 2011.
4. Nanex. Einstein and The Great Fed Robbery; 2013.
5. Angel JJ. When finance meets physics: The impact of the speed of light on financial markets and their regulation. Financial Review. 2014;49(2):271–281.
6. Einstein A. On the electrodynamics of moving bodies. 1905;.
7. Securities US, Commission E. Market Maker; 2000.
8. Menkveld AJ. High frequency trading and the new market makers. Journal of Financial Markets. 2013;16(4):712–740.
9. Securities US, Commission E. Regulation NMS; 2005.
10. Technologies A. Anova Technologies Network Map; 2018.
11. Securities US, Commission E. Regulation Alternative Trading System; 1998.
12. Securities US, Commission E. Regulation Alternative Trading System Amendments; 2015.
13. Securities US, Commission E. Measures to address market volatility; 2012.
14. Securities US, Commission E. Tick size pilot program; 2016.
