# Supplementary material for: Fragmentation and inefficiencies in US equity markets: Evidence from the Dow 30
Source: PLoS One. 2020 Jan 22;15(1):e0226968. doi: 10.1371/journal.pone.0226968 (PMC6975550; doi:10.1371/journal.pone.0226968)
Supplement: S2 Appendix — Provides formal definitions for many of the terms used in the study. (PDF) [file pone.0226968.s002.pdf]

2 Glossary

2.1 Market Architecture

Definition 1 (*Market System*)**.** *A market system may be defined as a network or graph which consists of a set of one or more market centers connected by a set of communication channels or (links), i.e.* $system = (centers,\ links)$*.*

Definition 2 (*Market Center*)**.** *A market center is a location, physical or digital, where agents may interact with a market system. A market center may be defined as a tuple containing a local order book, a set of valid actions, and a set of traded financial instruments, i.e.* $center = (book,\ actions,\ instruments)$*.*

Definition 3 (*Local Order Book*)**.** *The local order book contains information about the unfulfilled orders that have been submitted to a market center, allowing it to accumulate and maintain state. One possible representation of a local order book for a single financial instrument is two ordered lists of queues, where each list is associated with a side of the marked (bid/offer) and each queue is associated with a price.*

Definition 4 (*Action Set*)**.** *The action set defines the valid actions at a market center. No requirements are imposed on the action set, though a simple real world action set might allow for the submission of limit orders (which guarantee price), market orders (which guarantee execution), modification of resting orders, and cancellation of resting orders; i.e.* $actions = \{\text{limit order, market order, modify, cancel}\}$*.*

Definition 5 (*System Activity*)**.** *Let the system activity,* $\mathbb{A}$*, be a chronological list of all actions that are performed in a market system. This includes actions performed by market participants, administrative messages transmitted by regulators, and messages transmitted by the exchange(s).*

Definition 6 (*Data Feed*)**.** *A data feed,* D*, is defined to be any subset of the system activity of a market system (i.e.* $D \subseteq \mathbb{A}$*). Note that recorded occurrence times of identical events may vary between distinct data feeds due to physical considerations such as the finite speed of information propagation, desynchronized clocks, etc.*

2.2 Financial Instruments

Definition 7 (*Security*)**.** *A security is a financial instrument that represents partial or total ownership of an object or entity. Securities are fungible; securities belonging to the same "class" have the same value, and therefore are interchangeable. Additionally, the exact value of a security is negotiable. Common varieties of securities include stocks, bonds, and options, all of which may be traded on electronic markets, such as the NMS.*

Definition 8 (*Stock*)**.** *Stocks, which are also called equities or equity securities, are a variety of security that represents partial ownership of a publicly traded company. Stocks are a vehicle by which companies can acquire the capital necessary to grow and the secondary market for stocks is the basis of a large portion of the U.S. financial industry.*

2.3 The Best Bid/Offer

The following definitions assume the existence of a market system, $system = (centers,\ links)$. Each $center \in centers$ has an $action$ set that allows for limit orders and trades a financial instrument i. Additionally, there exists a data feed, D, that contains information about the top of the book at each market center (i.e., a consolidated quote feed).

Definition 9 (*Local Best Bid/Offer*). *The local best bid and offer (LBBO) is a tuple composed of the local best bid and the local best offer at a particular market center.*

The local best bid for i at a particular $center \in centers$, at a time, t, is given by the tuple (p, q), where p is the maximum price among all active bids for i in the book at center (as observed via data feed D) and q is the quantity of shares of i available at that price at center (i.e. $LBB(D, center, i, t) = (p, q)$). The local best offer is defined similarly, but uses the minimum price among active offers at center along with the number of shares associated with that order (i.e. $LBO(D, center, i, t) = (p', q')$).

Definition 10 (*Global Best Bid/Offer*). *The global best bid and offer (GBBO) is a tuple composed of the global best bid and the global best offer at a particular market center.*

The global best bid is similar to the local best bid, but is formed by the maximum price (and the quantity associated with that order) among resting bids for i among all market centers, i.e. $GBB(D, i, t) = (p'', q'')$. Similarly, the global best offer is formed by the minimum price among resting offers and the number of shares at that price (i.e. $GBO(D, i, t) = (p''', q''')$).

The NBBO, provided by the SIP, is an example of a GBBO in the NMS. Note that any real implementation of a GBBO necessitates the introduction of some amount of latency from propagation delays between the market centers and consolidating entity. This latency can have material implications in electronic markets where information propagation approaches the speed of light.

2.4 Market Inefficiencies

The following definitions assume the existence of a market system, $system = (centers,\ links)$, containing two market centers, two data feeds, D_1 and D_2, and a financial instrument i that is traded at each $center \in centers$. D_1 and D_2 are assumed to contain quote information from each market center, though they may have additional information that contributes to their uniqueness. Additionally, the distribution of reporting latency and timestamps associated with each event may differ between the feeds.

Note that these definitions are phrased for the best bid, but apply similarly to the best offer.

Definition 11 (*Price Discrepancy*). *A bid price discrepancy is said to occur when the best bid price differs between D_1 and D_2, i.e.*

$$\Delta BB(i, t) = BB(D_1, i, t).price - BB(D_2, i, t).price \neq 0.$$

Definition 12 (*Market Inefficiency*). *A market inefficiency occurs whenever a market participant is able to systematically profit from a price discrepancy, usually via the purchase and immediate sale of i.*

Definition 13 (*Dislocated Data Feeds*). *D_1 and D_2 are dislocated with respect to the best bid of i at a time t if there is a bid price discrepancy between D_1 and D_2.*

Definition 14 (*Dislocation*). *A dislocation between D_1 and D_2 occurs whenever they are dislocated with respect to the best bid of i over a half-open interval of time $[a, b)$.*

Definition 15 (*Differing trade*). *A trade is referred to as a differing trade if it occurs during the lifetime of a dislocation.*

Definition 16 (*Dislocation Segment*). *A dislocation segment with respect to the best bid of i is any half-open interval of time, $[a, b)$, where D_1 and D_2 are dislocated with respect to the best bid of i and $sgn(\Delta BB(i, t)) = sgn(\Delta BB(i, a))\ \forall t \in [a, b)$.*

Definition 17 (*Direction*)**.** *The direction of a dislocation segment over an interval* $[a, b)$ *is defined as* $sgn(\Delta BB(i, a))$*.*

Definition 18 (*Duration*)**.** *The duration of a dislocation or dislocation segment over an interval* $[a, b)$ *is defined as* $b - a$*.*

Definition 19 (*Magnitude*)**.** *The magnitude of a dislocation or dislocation segment over an interval* $[a, b)$ *may be defined as one of the following:*

$$max\_mag = \max_{t \in [a,b)} \{|\Delta BB(i,t)|\}$$
$$min\_mag = \min_{t \in [a,b)} \{|\Delta BB(i,t)|\}$$
$$mean\_mag = \frac{max\_mag + min\_mag}{2}$$

Definition 20 (*Realized Opportunity Cost*)**.** *The Realized Opportunity Cost (ROC) experienced by market participants over a period of time* $[a, b]$ *is defined as:*

$$\sum_{t \in T} |p_{D_1}(time(t), side(t)) - p_{D_2}(time(t), side(t))|,$$

where T *are all trades that occurred at the NBBO in the period* $[a, b]$*,* $time(t)$ *is a function that returns the time that trade* t *executed,* $side(\cdot)$ *returns the opposite side (bid or offer) of the order that instigated the trade,* $p_{D_1}(time, side)$ *returns the best price displayed on feed* D_1 *at the given time and on the given side, and* $p_{D_2}(time, side)$ *provides the same information for feed* D_2*.*

2.5 Market Actions

The following definitions provide a high-level description of the purpose and details of some common order types, but are not necessarily representative of implementations at NMS market centers.

Definition 21 (*Limit Order*)**.** *Guarantees market participants an execution price no worse than a provided limit price, but does not provide any guarantees about the timeliness of execution. This may be implemented by placing a received limit order into the price queue associated with the provided limit price on the correct side of the book (bid or offer, as specified by the order), assuming that it did not match with a resting order at a better price.*

Fields: Instrument identifier, bid/offer, limit price, desired quantity.

Definition 22 (*Market order*)**.** *Guarantees instant execution on a best effort basis, but does not provide any guarantees about the execution price. This may be implemented by matching the market order with the best resting orders on the opposite side of the book until the desired quantity is obtained. A market order may be thought of as a limit order with the limit price set in order to guarantee execution (i.e. 0 for a market offer or infinity for a market bid).*

Fields: Instrument identifier, bid/offer, desired quantity

Definition 23 (*Modify*)**.** *Allows market participants to update values associated with resting orders and allows for adaptation to changing market conditions. The main usage of this order is to change the number of shares required to fulfill a particular order, since modifying the limit price of order may cause it to lose its place in its current price queue.*

Fields: Order identifier, field(s) to modify, new value(s)

Definition 24 (*Cancel*)**.** *Allows market participants to remove resting orders from the local book prior to execution.*

Fields: Order identifier

Definition 25 (*Immediate Or Cancel*)**.** *Often shortened to IOC, this is a modifier which may be applied to any order rather than a stand alone order type. The modifier indicates that the associated order should be executed immediately upon receipt or canceled if immediate execution is not possible.*

Definition 26 (*Non-Displayed Orders*)**.** *Orders may be marked with a conditional flag which indicates that they should not be displayed on an exchanges order book, in part or whole. Such orders are sometimes referred to as hidden orders, since market participants can not identify active non-displayed orders in an order book from publicly available information.*

Non-displayed orders may come with some negative consequences including increased fees and decreased execution priority in comparison with displayed orders with identical attributes.

Definition 27 (*Midpoint Peg*)**.** *A variety of hidden order that executes at the midpoint of the NBBO, i.e.* $0.5(NBB.price + NBO.price)$.
