# Supplementary material for: Fragmentation and inefficiencies in US equity markets: Evidence from the Dow 30
Source: PLoS One. 2020 Jan 22;15(1):e0226968. doi: 10.1371/journal.pone.0226968 (PMC6975550; doi:10.1371/journal.pone.0226968)
Supplement: S3 Appendix — A high level summary of some regulations that impact the implementation of the National Market System in the US. (PDF) [file pone.0226968.s003.pdf]

3 Regulation National Market System

Regulation National Market System (Reg. NMS) is the set of regulations which defines much of the macro-level organization of the U.S. NMS. The primary goal of Reg. NMS is the creation a unified National Stock Market, additionally it has two secondary goals: to promote competition between markets and between orders, and to serve the interests of long-term investors and listing companies [1]. Reg. NMS is composed of several rules and regulations, the most important of which are summarized below. See [1] for more details.

3.1 Order Protection Rule

The Order Protection Rule (Rule 611), also known as the Trade-through Rule, is meant to protect orders from trade-throughs, which occur when a market center matches an order against a local counter-party when a better price is available via a protected quotation displayed by an alternative market center. Note that a "better" price in this context is defined from the perspective of the new order entering the market, a.k.a. a liquidity demanding or liquidity consuming order. Therefore a lower execution price is be considered better for an entering bid (offer to buy), while a higher execution price is be considered better for an entering offer (offer to sell).

A protected quotation is defined in Reg. NMS as a bid or offer quotation that satisfies the following properties: the quotation must be automated, the quotation must be displayed by an automated trading center, and the quotation must offer the lowest offer price or highest bid price among all publicly displayed quotations.

A quotation is considered automated if it may be executed without human intervention (up to the full listed quantity), allows for the correct execution of Immediate-Or-Cancel (IOC) orders against the quotation, immediately provides a response to the sender of an Immediate-Or-Cancel order indicating the execution status of that order, and immediately updates the quotation to reflect any changes to its status.

A trading center is considered automated if it implements systems and procedures that allow it to display automated quotations as defined above, and quotations that do not satisfy the requirements of an automated quotation are identified as manual quotations as quickly as possible.

Trade-throughs are prohibited Under Rule 611, however exceptions are allowed for Inter-market Sweep Orders (ISO), quotations displayed by markets that fail to meet the reporting requirements for automated quotations, and flickering quotations with multiple prices displayed in a single second.

3.2 Access Rule

The Access Rule (Rule 610) concerns itself with setting standards for access to quotations in NMS stocks, and caps the fees that an exchange may charge for accessing its protected quotations at $0.003 per share. Rule 610 allows for the creation and usage of private data feeds, often referred to as direct feeds by market participants since they are offered directly by exchanges rather than through a third party. Rule 610 also prohibits trading centers from displaying quotations which would lock or cross a protected quotation from a different trading center.

A market is said to be locked if the bid-offer spread of that market is zero, in other words there exists a resting bid and a resting offer with identical limit prices. A market is said to be crossed if the bid-offer spread of that market is negative, i.e. there exists a resting bid whose limit price is greater than the limit price of a resting offer, or equivalently a resting offer exists whose limit price is less than the limit price of a

resting bid. These effects are the result of coupling geographically fragmented exchanges, since an order that may lock or cross a market would immediately find a counter-party if the two orders were present on the same exchange.

3.3 Sub-Penny Rule

The Sub-Penny Rule (Rule 612) prohibits market participants from displaying or accepting quotations for NMS stocks priced in an increment less than $0.01 unless the quotation price is less than $1.00, in which case the minimum increment is $0.0001. Rule 612 is meant to prohibit the practice of "sub-pennying" in which market participants could "step ahead" of a protected quotation by providing a negligible amount of price improvement, allowing the "sub-pennied" order faster execution at effectively no extra cost.

The significance of this rule, with respect to geographic fragmentation and market inefficiencies, is that the minimum increment for the quoted price of a traded instrument sets the minimum magnitude of all dislocation segments.

3.4 Market Data Rules

Rules 601 and 603 are referred to as Market Data Rules and are meant to promote wide availability of market data, thus providing all market participants with an accurate and reliable source of information on the best prices in NMS stocks. These rules cover the organization of a consolidated data feed for NMS stocks, the reward structure for contributing information to the consolidated data feed, and establishes standards for quote and trade information provided to and provided by the consolidated data feed.

In particular these rules concern the Consolidated Tape Association (CTA) plan which disseminates transaction information for NYSE listed securities, the Consolidated Quotation (CQ) plan which disseminates quote information for NYSE listed securities, and the Nasdaq UTP plan which disseminates quote and trade data for Nasdaq listed securities. The information provided by the CTA plan and CQ plan forms Consolidated Tape A, and the information provided by the UTP plan forms Consolidated Tape C. There also exists a Consolidated Tape B which reports trade information for stocks listed on regional exchanges. The aggregation of Consolidated Tapes A, B, and C form what is commonly referred to as the SIP feed.

References

1. Securities US, Commission E. Regulation NMS; 2005.
