# Supplementary material for: Fragmentation and inefficiencies in US equity markets: Evidence from the Dow 30
Source: PLoS One. 2020 Jan 22;15(1):e0226968. doi: 10.1371/journal.pone.0226968 (PMC6975550; doi:10.1371/journal.pone.0226968)
Supplement: S4 Appendix — Additional details on how we calculate Realized Opportunity Cost and Dislocations. Also provides some example calculations and discussion regarding the connections between ROC and Dislocations. (PDF) [file pone.0226968.s004.pdf]

4 Dislocations and ROC

4.1 Calculating realized opportunity cost

Calculating Realized Opportunity Cost (ROC) For each trade of interest: Obtain the Securities Information Processor (SIP) National Best Bid and Offer (NBBO) prices and the Direct Best Bid and Offer (DBBO) at the time of the trade. Check if the trade executed at one of the NBBO prices. If yes, then the difference between the execution price and the corresponding price from the DBBO, multiplied by the number of shares transacted, becomes the ROC associated with that trade. Note: Depending on the side of the active order (bid or offer), and the relationship between the NBBO and DBBO, the ROC may be identified as favoring the SIP or a Direct feed. In other words, when the active order could receive price improvement by executing at the price displayed by the DBBO, then the ROC becomes associated with the SIP (SIP ROC). Likewise, if the active order received a price improvement by executing at the NBBO rather than the DBBO, then the ROC becomes associated with the Direct feeds (Direct ROC). If no, then the trade is discarded from the analysis, since it is difficult to accurately determine the side of the active order in this situation—and knowing the side of the active order is required in order to accurately calculate the directional ROC. Note: ROC experienced on both sides of the book (bid and offer) are aggregated over each day, ticker, and exchange; thus, there may be some cancellation between positive ROC (Direct ROC) and negative ROC (SIP ROC) during the aggregation to determine net ROC for that day-ticker-exchange. The net ROC is therefore a conservative measure, since it is possible that investors could experience both SIP and Direct ROC for that day-ticker-exchange.

4.2 Example

In particular, see the 79th trade in S3 Table, where 100 shares of AAPL transacted at \$99.13 at 9:48:55.398386. The NBBO at that time was (bid @ \$99.13, offer @ \$99.15), while the DBBO was (bid @ \$99.16, offer @ \$99.17). Since the trade executed at \$99.13, the best bid displayed by the SIP, we infer that the resting order was a bid and the active order was an offer. The ROC is then calculated as (\$99.13 per share - \$99.16 per share) * 100 shares = (-\$0.03 per share) * 100 shares = -\$3.00 in favor of the Direct feeds (i.e. SIP ROC). From this example, one can note that when the active order is an offer, then the formula for ROC is (SIP National Best Bid (NBB) - Direct Best Bid (DBB)) * shares. This results in a positive value when the NBO provides price improvement for the active bid and a negative value when the DBO provides price improvement for the active bid. Additionally, see the 95th trade in S3 Table where 100 shares transacted at \$99.14 at 9:48:55.398560. The NBBO at that time was (bid @ \$99.14, offer @ \$99.14) and the DBBO was (bid @ \$99.16, offer @ \$99.17). Since the SIP was locked at the time of execution the active order could have been from either side of the book. For this example, we will focus on the situation where we assume the active order is a bid and the resting order is an offer. The ROC is then calculated as (\$99.17 per share - \$99.14 per share) * 100 shares = (\$0.03 per share) * 100 shares = \$3.00 in favor of the SIP (i.e. Direct ROC). Note that in this example, the formula used to calculate the ROC reverses the position of the SIP and Direct prices since the active order is a bid instead of an offer. Thus, the formula for ROC is (Direct Best Offer (DBO) - SIP Best Offer (NBO)) * shares. This maintains the meaning of the sign, where positive values indicate price improvement featured by the NBB and negative values indicate price improvement featured by the DBB (from the perspective of the active order). Thus, ROC from both sides of the book may be treated uniformly in that positive values favor the SIP feed and negative values favor the consolidated Direct

feeds. We aggregate the ROC by date, stock, and venue. Since these two trades occurred at the same trading venue, they would be summed, resulting in a net ROC of $0.00. Similar cancellations occur for every date-stock-venue combination resulting in these conservative measures of ROC. Additionally, in the example dislocation there were almost 100 differing trades (i.e., trades that occurred while the NBBO and DBBO are dislocated) as contained in S3 Table. Yet, our ROC measures only include trade executions at the NBBO. Therefore, we only consider a total of 11 trades, 6 with positive ROC (see S4 Table) and 5 with negative ROC (see S5 Table), during this dislocation, thus providing additional evidence that our ROC measures are conservative.

4.3 Dislocations

In Fig. 7 from the main body, we see all dislocations in AAPL on January 7, 2016. We select an arbitrary dislocation to investigate which existed on the offer side from 9:48:55.396886 to 9:48:55.398749 (a duration of 1863 microseconds). This dislocation features a maximum value of $0.06, which occurs between 9:48:55.397644 and 9:48:55.398027 (a duration of 383 microseconds or 20.56% of its lifetime). During this time where the dislocation featured its maximum value, the SIP best offer remained at $99.11 and the Direct best offer remained at $99.17. Any bid orders submitted during this period stood to save $0.06 per share by transacting at the SIP BO rather than the Direct BO, assuming that they could actually locate resting offers at $99.11, either in the lit or dark markets. Note that this dislocation started and ended while the Limit-up Limit-down (LULD) mechanism was in effect (this is engaged at 9:45 each day, following the first 15 minutes of trading), featured a duration longer than 545 microseconds (what we consider to be the minimum duration in order to be actionable) and featured a maximum magnitude greater than $0.01. Note: you can find more info on LULD here: http://www.luldplan.com/index.html.

4.4 Connecting ROC and Dislocations

The ROC statistic captures events that occurred (i.e. trades) and assigns an opportunity cost to them based on the state of the SIP and Direct feeds at the time of the trade. Hopefully the above example has illustrated the extreme sparsity of our ROC approach, which only considers trades that execute at either side of the prevailing NBBO, features cancellation effects due to aggregation, and does not consider duration/actionability (e.g., could the agent who entered the active order have reasonably reacted to the state of the two feeds?). Dislocation segments are constructed to capture the relative states between the NBBO and DBBO through time, observing the dislocations between the two feeds and collecting information about their duration and magnitude. With our approach, we capture the inefficiencies and opportunity costs that actually occurred (i.e., realized), and what inefficiencies and opportunity costs could have occurred (i.e., dislocation segments). For illustrative purposes only, if the NBBO and DBBO were tightly synchronized, then the ROC statistic would tend towards $0.00. [Note: there are specific policy reasons in Reg. NMS that SIP reporting will always lag reporting on the direct feeds, independent of technological infrastructure]. Thus, constructing DSs so that they only consider the NBBO and DBBO allows us to isolate one component of the ROC statistic and investigate it in greater detail. Additionally, ROC does not account for duration/actionability, while DSs allow for such considerations to be addressed in a simple and direct way. These two measurements, ROC and DSs, were constructed to investigate similar phenomena from slightly different perspectives to provide complementary and synergistic views of NMS dynamics.

References

1. Bartlett RP, McCrary J. How rigged are stock markets? Evidence from microsecond timestamps. Journal of Financial Markets. 2019;.

S3 Table. Example AAPL Trades. Trades that occurred during a dislocation in AAPL on 2016-01-07 at approximately 9:48am, more than three minutes after the trading "guardrails" are enforced. The "Delta" column indicates the difference between the Thesys timestamp and the SIP publication timestamp (in microseconds). For trade 0, Thesys received the trade at 9:48:55.396951 and the SIP timestamp was 9:48:55.396696. The "Extra" column contains additional deltas related to the timestamps added in the 2015 SIP changes, see [1] for additional details. In particular, this column contains the difference (in microseconds) between the Thesys timestamp and the exchange timestamp. For trade 0, Thesys received the trade at 9:48:55.396951 and the exchange timestamp was 9:48:55.397602, an example of the timestamp inversion seen in [1], which is generally cause by clock synchronization issues.

S4 Table. Example AAPL Trades with Positive ROC. A subset of the trades from Table S3 that resulted in positive ROC. Positive ROC indicates that these trades received favorable prices that were aligned with the SIP NBBO.

S5 Table. Example AAPL Trades with Negative ROC. A subset of the trades from Table S3 that resulted in negative ROC. Negative ROC indicates that these trades executed at less favorable prices than what was offered by the DBBO.
