# Supplementary material for: Fragmentation and inefficiencies in US equity markets: Evidence from the Dow 30
Source: PLoS One. 2020 Jan 22;15(1):e0226968. doi: 10.1371/journal.pone.0226968 (PMC6975550; doi:10.1371/journal.pone.0226968)
Supplement: S1 Table — Aggregated by day and trading symbol. (PDF) [file pone.0226968.s005.pdf]

Symbol		Trades	Traded Val	Diff Trades	Diff Traded Val	ROC	ROC/Share
AAPL	mean	174,820.85	2,542,188,952.00	34,316.58	483,265,898.89	45,852.81	0.007569
	std	68,897.09	1,040,923,482.82	20,556.77	280,321,422.73	27,275.35	0.001224
	min	54,824	983,856,430.54	2,112	35,009,317.38	2,773.35	0.004007
	25%	129,830	1,872,512,861.35	23,498.75	340,459,129.85	32,088.96	0.007307
	50%	156,198.50	2,272,037,106.11	32,741	452,246,993.35	43,204.10	0.007682
	75%	199,793.25	2,870,019,105.28	42,674.75	599,146,631.96	57,647.05	0.008351
	max	517,270	8,280,915,338.59	103,885	1,596,912,962.05	138,331.08	0.011924
AXP	mean	32,348.46	250,614,304.97	9,086.69	71,464,081.61	11,622.14	0.008811
	std	16,110.77	143,031,721.64	4,434.64	36,283,858.01	7,156.73	0.000757
	min	11,095	90,438,986.65	2,219	19,241,382.52	2,666.91	0.007285
	25%	22,756.50	168,209,590.34	5,999.50	49,149,197.52	7,672.38	0.008324
	50%	26,835	207,178,850.49	7,476	57,481,058.84	8,987.37	0.008723
	75%	37,067.75	277,456,051.09	10,905.75	86,485,488.61	13,792.36	0.009248
	max	159,135	1,468,245,304.80	31,507	302,294,385.78	75,473.73	0.013393
BA	mean	20,749.26	288,851,358.58	7,071.25	100,312,506.47	10,955.15	0.012812
	std	10,435.29	154,859,396.19	3,027.87	41,626,820.43	6,235.13	0.008003
	min	4,220	60,869,511.81	1,209	22,712,059.97	2,404.84	0.008497
	25%	14,825.75	202,629,761.69	4,865	69,859,447.55	6,607.66	0.010588
	50%	18,904	260,864,798.97	6,613.50	95,165,851.81	9,608.32	0.011730
	75%	24,641.25	339,733,518.51	8,877.75	123,131,081.82	13,061.48	0.013252
	max	101,159	1,496,951,020.26	19,630	303,000,376.46	47,010.92	0.131181
CAT	mean	30,586.73	269,579,023.84	9,239.74	81,143,988.26	11,986.17	0.010142
	std	11,384.23	107,296,519.47	3,721.88	30,953,831.34	8,988.83	0.005617
	min	7,660	72,342,016.91	2,283	24,025,499.19	2,847.50	0.007044
	25%	22,670.50	204,956,948.21	6,684.75	58,633,048.01	7,680.36	0.008842
	50%	28,267	245,802,664.58	8,451	76,013,433.31	10,301.82	0.009394
	75%	36,304.25	323,347,949.42	10,730	95,123,308.69	13,455.58	0.010105
	max	77,886	964,799,514.35	22,381	222,261,612.89	100,244.92	0.084153
CSCO	mean	77,364.30	493,693,519.98	11,555.12	74,134,548.33	26,409.30	0.008899
	std	33,235.82	207,062,395.07	8,173.36	50,695,319.75	19,401.61	0.001000
	min	31,865	182,535,557.30	660	4,502,758.25	1,461.77	0.005896
	25%	58,015	367,489,467.23	6,881	46,381,850.87	15,394.81	0.008321
	50%	68,328.50	444,190,912.86	10,643	70,264,638.23	23,922.43	0.009015
	75%	86,368.50	548,980,902.84	14,364.50	92,558,544.11	32,439.85	0.009212
	max	307,808	1,702,786,754.09	58,922	316,907,129.91	130,317.79	0.019144
CVX	mean	44,441.79	462,460,384.39	12,439.81	134,648,014.78	17,036.07	0.012786
	std	17,816.08	164,739,606.44	6,693.09	56,874,558.81	16,228.50	0.034249
	min	13,879	144,582,207.22	2,377	28,830,654.89	2,456.15	0.006135
	25%	32,594.50	346,722,417.91	8,344.75	97,784,127.50	9,772.81	0.008240
	50%	39,655.50	430,819,298.93	10,794.50	122,659,276.18	12,993.21	0.008796
	75%	53,123.50	538,846,282.98	14,257.25	158,567,573.75	18,798.64	0.009602
	max	148,515	1,263,782,534.87	50,186	423,871,063.95	190,901.32	0.531465
DD	mean	18,036.06	132,521,012.09	4,913.74	37,476,052.09	6,342.15	0.009882
	std	8,759.67	63,295,360.33	2,764.13	19,045,796.05	4,403.69	0.004642
	min	5,262	40,582,912.43	773	6,491,584.69	832.8600	0.004446
	25%	12,017	89,557,470.32	3,123.50	24,611,595.27	3,912.60	0.008929
	50%	15,462	114,690,819.55	4,104.50	32,687,710.05	5,066.52	0.009511
	75%	20,793.50	155,332,165.60	5,499	44,016,133.46	7,243.21	0.010036
	max	52,298	418,605,566.86	15,217	113,435,890.12	42,392.91	0.080300
DIS	mean	41,156.78	495,392,306.10	10,535.97	129,495,234.75	39,331.64	0.024431
	std	15,686.14	208,901,188.83	4,550.24	53,409,308.00	323,220.91	0.153256
	min	17,030	203,854,389.74	2,633	36,156,641.31	3,221.94	0.006826
	25%	31,892.25	374,803,933.38	7,657.50	97,517,442.34	10,284.53	0.008200
	50%	36,745.50	430,039,189.49	9,220.50	114,691,273.09	13,055.17	0.008814
	75%	45,623.25	558,118,900.42	11,989	144,599,501.14	17,818.25	0.010073
	max	124,145	1,659,028,038.95	32,212	369,007,239.36	5,138,897.26	2.4261
GE	mean	83,963.26	741,830,493.33	12,828.05	119,789,470.34	44,606.60	0.011460
	std	35,661.52	309,010,418.40	8,012.21	69,234,760.11	71,027.27	0.009108
	min	27,905	290,466,991.56	2,653	33,035,264.68	7,844.38	0.005032
	25%	59,365.25	514,268,974.25	7,603	74,660,777.81	23,089.88	0.008105
	50%	74,767.50	670,261,948.27	10,589	96,944,717.80	29,655.10	0.009088
	75%	96,517	876,527,780.45	14,826	141,143,821.44	45,447.71	0.009994
	max	236,395	1,961,985,442.37	49,675	427,596,291.36	1,020,533.87	0.074863

Continued on next page

Symbol		Trades	Traded Val	Diff Trades	Diff Traded Val	ROC	ROC/Share
GS	mean	16,072.52	266,630,735.82	6,039.60	100,455,871.70	12,632.51	0.018917
	std	6,759.46	124,491,943.62	2,299.09	38,813,844.47	7,817.49	0.008519
	min	5,914	106,821,197.38	1,908	43,864,040.44	4,126.93	0.009892
	25%	11,672.50	178,117,450.68	4,400.50	72,797,279.72	8,094.88	0.015293
	50%	14,285	224,601,809.66	5,593.50	89,806,560.53	10,478.40	0.017610
	75%	18,995.50	329,800,789.60	7,207.25	123,468,835.06	14,081.00	0.020349
	max	50,816	857,877,495.97	14,393	247,177,637.53	72,612.29	0.117195
HD	mean	27,728.62	366,840,862.69	8,920.89	123,442,984.04	12,744.17	0.011027
	std	8,963.39	127,799,636.38	3,551.59	45,816,593.86	13,953.86	0.005344
	min	13,006	165,434,810.90	2,515	36,439,575.01	2,864.63	0.007334
	25%	21,668.50	276,025,739.12	6,473	92,772,210.99	7,812.18	0.009211
	50%	25,747	339,935,686.44	8,234	115,952,305.04	9,863.20	0.009837
	75%	31,341.50	416,697,720.23	10,762	146,870,527.07	13,201.84	0.010717
	max	64,114	1,031,531,952.92	22,597	291,592,154.20	186,403.78	0.059372
IBM	mean	19,503.60	283,053,487.10	6,540.58	97,629,157.53	10,322.91	0.023045
	std	7,762.60	121,204,978.80	3,031.09	41,935,403.95	11,852.61	0.155151
	min	6,168	83,951,134.35	1,493	24,252,638	2,042.64	0.007853
	25%	14,595	209,597,644.74	4,586	71,732,705.50	5,972.78	0.010419
	50%	17,729	252,167,134.97	5,852.50	89,826,517.09	7,844.79	0.011260
	75%	22,431.25	328,204,236.66	7,532.75	111,719,286.16	10,385.29	0.012460
	max	59,625	972,131,459.03	21,810	299,050,973.50	111,628.46	2.4712
INTC	mean	88,012.92	539,061,461.61	13,623.27	80,485,200.80	24,652.76	0.008581
	std	32,133.18	218,280,102.40	8,604.73	50,349,950.02	16,048.53	0.001366
	min	25,392	174,808,926.57	668	3,512,129.76	906.3800	0.003979
	25%	66,319.50	409,452,090.75	8,564.50	53,076,046.07	15,370.02	0.008123
	50%	81,767	493,100,646.52	13,526	79,046,604.94	23,962.08	0.008955
	75%	100,219.25	601,791,580.25	17,608.50	104,796,930.94	32,243.13	0.009165
	max	233,578	1,765,833,707.79	48,079	318,483,188.44	91,380.43	0.017641
JNJ	mean	41,248.16	516,784,968.61	10,117.01	132,739,127.27	15,971.14	0.011066
	std	13,010.19	163,195,302.28	4,751.53	54,033,725.20	24,562.10	0.009799
	min	15,606	194,794,413.45	2,156	34,113,674.01	3,046.94	0.006887
	25%	32,847.50	413,846,348.61	7,231.25	98,042,130.74	8,347.87	0.008033
	50%	38,411.50	483,292,741.16	8,718	117,582,458.80	10,975.76	0.008545
	75%	45,961.50	586,813,347.06	11,288	153,593,921.79	16,623.58	0.009356
	max	94,603	1,244,615,527.23	32,165	338,562,051.69	362,771.34	0.091514
JPM	mean	88,003.57	801,423,694.85	21,356.75	193,852,644.59	29,550.37	0.008671
	std	39,466.22	360,958,601.04	11,483.72	91,730,373.77	14,749.77	0.001427
	min	30,040	331,806,293.97	4,953	58,788,624.46	7,089.25	0.006291
	25%	61,325.75	565,821,050.04	13,638	130,610,914.09	19,065.50	0.007994
	50%	77,139	711,684,130.50	17,913.50	171,373,698.77	25,663.01	0.008471
	75%	101,690.75	948,789,239.22	25,153	232,200,018.53	34,390.20	0.008981
	max	256,973	3,004,137,079.38	70,052	646,651,792.53	92,386.71	0.019550
KO	mean	52,120.74	406,264,869.51	10,086.25	81,371,474.40	18,263.90	0.009458
	std	19,287.46	161,269,975.11	4,577.72	33,628,799.23	8,429.36	0.003791
	min	19,958	185,384,176.07	3,156	30,732,830.23	7,111.74	0.006435
	25%	39,138.50	301,353,437.57	7,209.25	59,076,462.53	13,153.95	0.008509
	50%	47,536.50	368,857,020.57	8,995	74,612,460.50	16,482.05	0.008996
	75%	58,796	463,324,316.41	11,326.50	92,283,870.91	20,688.83	0.009567
	max	151,901	1,308,364,552.46	30,895	222,649,014.88	88,890.33	0.059954
MCD	mean	28,809.30	380,847,318.26	7,442.77	103,499,997.57	10,822.55	0.010045
	std	9,250.20	146,529,362.71	2,681.91	39,288,571.37	10,847.35	0.004427
	min	9,911	117,553,924	2,479	32,522,381.94	2,926.51	0.007534
	25%	22,526.25	277,305,454.74	5,422.50	75,412,153.02	6,484.23	0.008638
	50%	26,999.50	355,968,666.10	7,088.50	98,050,825.62	8,795.32	0.009242
	75%	33,173.25	455,898,847.39	8,601.50	121,862,623.83	11,289.40	0.009876
	max	72,028	1,044,773,633.09	20,018	265,940,261.14	114,279.57	0.055288
MMM	mean	11,365.37	167,307,657.17	3,636.52	57,734,183.69	12,063.44	0.017206
	std	3,901.98	56,357,516.03	1,769.07	23,315,655.68	102,963.80	0.055266
	min	3,704	42,376,029.54	852	12,737,335.17	1,268.98	0.008572
	25%	8,870.50	128,614,072.44	2,564	42,198,399.30	3,302.92	0.011656
	50%	10,484	156,620,977.62	3,148	53,116,097.50	4,412.75	0.012771
	75%	13,011	192,588,435.88	4,113	67,265,920.46	6,182.75	0.014374
	max	27,168	374,180,512.59	11,339	141,420,561.60	1,638,916.42	0.888354

Continued on next page

Symbol		Trades	Traded Val	Diff Trades	Diff Traded Val	ROC	ROC/Share
MRK	mean	52,065.45	404,241,094.10	12,269.51	97,773,420.29	17,435.31	0.008974
	std	21,247.82	198,964,179.96	6,450.80	49,864,763.12	10,578.05	0.002336
	min	18,727	139,953,296.94	4,541	37,156,365.39	5,935.82	0.005243
	25%	39,157.50	302,275,577.81	7,789	64,970,853.90	10,991.95	0.008151
	50%	46,619.50	360,181,487.45	10,518	84,618,791.63	15,107.30	0.008574
	75%	58,293.50	460,791,595.64	13,950.25	113,701,288.72	19,811.20	0.008998
	max	232,717	2,584,131,245.57	46,595	456,348,016.09	112,089.37	0.027925
MSFT	mean	141,856.07	1,190,901,402.50	24,761.04	203,129,267.74	36,706.48	0.008303
	std	63,588.22	533,057,860.34	17,480.90	139,593,661.04	25,629.62	0.001006
	min	37,036	459,917,664.02	1,070	8,596,209.02	1,253.33	0.004649
	25%	102,602.75	837,915,412.53	14,050.50	122,690,779.06	22,117.84	0.007944
	50%	124,327.50	1,053,837,918.53	22,263	180,976,495.62	32,474.83	0.008414
	75%	156,482	1,373,674,878.44	32,070.75	262,037,990.81	48,649.14	0.008943
	max	456,106	4,125,126,448	98,307	950,946,403.87	138,913.71	0.010702
NKE	mean	46,386.10	377,535,172.78	10,935.36	89,164,054.37	18,227.11	0.009535
	std	15,357.30	145,806,631.19	3,796.12	32,750,534.77	20,652.07	0.006259
	min	13,818	84,721,641.01	2,885	18,676,669.13	3,523.46	0.005848
	25%	37,737.50	295,226,871.17	8,613.25	69,144,244.49	12,031.69	0.008070
	50%	42,544	344,601,219.52	9,822	80,592,736.50	14,390.93	0.008480
	75%	51,532.50	424,862,753.77	12,534	102,100,476.22	18,212.90	0.008969
	max	121,962	1,195,681,284.35	28,410	232,923,873.16	280,266.40	0.084753
PFE	mean	91,040.68	692,324,391.87	13,862.73	110,715,986.10	31,625.70	0.009084
	std	49,256.08	473,362,104.74	6,672.49	60,406,629.99	16,222.08	0.002189
	min	32,599	212,898,806.65	4,422	28,855,501.39	8,447.34	0.005328
	25%	59,097.50	426,001,630.46	9,726.75	74,658,424.30	21,093.39	0.008060
	50%	80,628	611,356,656.02	13,270.50	103,824,254.59	29,745.09	0.008872
	75%	109,044.50	783,454,707.50	16,379	129,458,335.77	36,810.46	0.009218
	max	474,221	5,427,524,575.47	56,238	602,885,333.66	145,936.99	0.021475
PG	mean	50,438.27	570,844,223.65	11,760.85	134,139,256.91	17,786.87	0.011319
	std	26,464.80	419,733,122.26	5,828.25	70,501,433.31	13,441.76	0.027016
	min	19,980	185,431,171.67	3,696	40,926,831.50	4,789.61	0.005871
	25%	34,682.50	362,299,048	7,530.75	87,831,864.68	10,158.57	0.007830
	50%	43,215.50	456,219,304.50	10,168.50	113,664,173.39	14,134.84	0.008337
	75%	57,796	612,257,033.06	14,163	160,102,759.35	20,335.25	0.008869
	max	181,697	3,330,428,860.98	38,467	460,594,145.16	111,040.42	0.427532
TRV	mean	10,544.19	106,389,400.10	3,568.88	39,506,286.77	4,441.92	0.011206
	std	3,416.58	36,241,051.32	1,447.62	15,393,415.99	2,794.22	0.002964
	min	3,018	27,592,851.46	771	7,628,101.68	964.9800	0.007730
	25%	8,487.25	82,492,360.44	2,705	29,702,903.75	2,990.61	0.009813
	50%	9,965.50	101,071,670.21	3,334.50	37,475,398.81	3,837.38	0.010699
	75%	12,010.25	122,831,584.80	4,172.25	46,650,233.97	4,933.57	0.011895
	max	27,468	294,476,802.95	11,339	107,591,813.81	28,594.17	0.048296
UNH	mean	17,446.67	228,660,097.56	5,642.65	77,377,042.02	7,680.73	0.011369
	std	5,246.70	81,435,935.28	2,011.09	27,032,487.15	4,216.99	0.001956
	min	6,412	89,234,548.68	1,849	26,225,274.13	2,378.89	0.008077
	25%	14,129	173,512,633.11	4,413.50	59,089,553.72	5,357.03	0.010139
	50%	16,636	214,637,619.41	5,371.50	75,046,042.50	6,539.35	0.010909
	75%	19,932.50	260,900,529.94	6,717.75	92,546,473.56	8,912.00	0.012157
	max	41,842	725,532,688.10	15,652	218,550,591.96	30,826.07	0.020873
UTX	mean	24,903.26	263,375,122.23	8,217.23	88,158,366.16	17,510.92	0.011823
	std	12,739.37	141,586,417.29	4,913.50	47,017,041.73	109,897.32	0.025491
	min	5,358	49,595,310.95	1,315	13,549,323.03	1,579.70	0.007425
	25%	16,977	182,655,118.69	4,942.75	59,862,917.81	6,260.83	0.009197
	50%	21,463	229,710,235.45	7,034.50	77,766,133.91	8,629.70	0.009769
	75%	27,806.50	295,642,614.43	9,447.25	101,084,369.56	11,780.77	0.010558
	max	86,284	1,144,629,181.20	29,297	275,444,139.17	1,749,683.12	0.413092
V	mean	48,950.33	460,497,961.48	13,097.62	122,925,302.52	16,818.68	0.009524
	std	17,793.66	170,963,876.45	6,162.95	50,951,708.77	9,603.52	0.007991
	min	23,142	162,781,451.21	3,273	30,311,313.92	3,873.41	0.005686
	25%	36,797	351,926,962.18	9,092.75	88,092,316.21	11,157.63	0.007977
	50%	44,660	411,627,578.41	11,552	112,507,821.59	14,624.21	0.008478
	75%	56,347	531,962,904.82	14,735	139,117,677.04	18,457.99	0.009089
	max	128,775	1,261,830,529.49	42,661	355,125,487.75	85,584.79	0.120466

Continued on next page

Symbol		Trades	Traded Val	Diff Trades	Diff Traded Val	ROC	ROC/Share
VZ	mean	62,098.01	494,149,523.80	13,525.58	109,544,287.76	51,450.08	0.013465
	std	23,339.73	185,520,474.01	5,963.55	44,308,281.09	427,124.11	0.030589
	min	29,671	204,408,079.42	5,039	41,836,595.67	6,539.31	0.005940
	25%	46,137.50	362,469,762.67	9,445.50	77,277,337.22	14,070.98	0.008312
	50%	55,823.50	449,943,857.51	11,922	100,842,957.95	19,546.38	0.008925
	75%	71,345	574,383,307.99	15,340.50	128,753,322.82	27,179.16	0.009833
	max	147,919	1,264,130,771.03	36,340	266,067,716.19	6,798,041.07	0.469146
WMT	mean	49,823.30	448,218,124.11	11,786.63	109,524,107.26	19,815.12	0.011010
	std	20,042.63	187,614,765.33	5,642.63	49,138,231.17	26,412.77	0.013753
	min	20,706	211,540,076.99	3,709	34,219,678.86	4,605.33	0.006303
	25%	36,156.25	325,522,820.91	7,935	74,826,489.33	10,770.16	0.008199
	50%	44,622.50	399,048,171.16	10,657	99,148,394.58	14,630.73	0.008728
	75%	57,546.50	520,989,325.68	13,105.25	125,786,171.91	19,936.97	0.009294
	max	156,021	1,562,166,750.41	36,698	361,429,655.92	246,675.56	0.176158
XOM	mean	64,074.02	670,862,447.91	17,774.64	188,657,442.78	35,104.83	0.013337
	std	28,483.97	265,569,760.30	10,924.37	93,450,720.08	127,162.28	0.021539
	min	21,646	201,555,090.63	4,205	46,296,094.35	4,953.61	0.005362
	25%	46,888	496,895,704.13	11,816.25	129,373,837.81	15,072.46	0.007813
	50%	55,080.50	593,690,988.09	14,020	162,976,539.44	18,862.46	0.008369
	75%	74,045.75	786,147,285.48	19,397.50	211,792,668.60	31,372.43	0.010116
	max	209,816	1,761,362,028.61	75,421	613,405,517.24	2,003,841.58	0.238129

S1 Table. Summary ROC Statistics for Dow 30 Stocks. Aggregated by day and trading symbol.
