# Supplementary material for: Fragmentation and inefficiencies in US equity markets: Evidence from the Dow 30
Source: PLoS One. 2020 Jan 22;15(1):e0226968. doi: 10.1371/journal.pone.0226968 (PMC6975550; doi:10.1371/journal.pone.0226968)
Supplement: S2 Table — The pricing presented in this table assumes a single consumer with an academic use case aiming to construct a dataset similar to what was used in this analysis. It is also assumed that non-display fees do not apply. Historical data costs assume a 12 month period of interest, i.e. calendar year 2016. Strictly speaking, historical data may sufficient for replicating the analysis presented in this paper, making subscription to live feeds unnecessary. However, utilizing historical data provided by each exchange excludes the possibility of collecting data from a single point of observation, reintroducing the issues of clock synchronization and relativity. Additionally, highlighting the monthly cost for comprehensive direct feed access shines a light on one of the reasons for the lack of academic participation in the analysis of modern U.S. stock markets. This does not include costs which may be incurred while curating the data, fulfilling potential co-location requirements, ISP/networking costs, computing hardware acquisition and maintenance, etc. DoB indicates that a product contains full Depth of Book information (adds, mod, and cancel messages), while ToB indicates that a product contains only Top of Book information (trade and quote messages). The NYSE Historical ToB product, also called NYSE Daily TAQ, is frequently used in academic studies due to it’s relatively low cost and broad coverage (e.g. [24] use this product). Historical data from CHX is not directly available, and the live feeds are transitioning to NYSE technology, thus historical CHX data must be purchased from a third party. This list is not guaranteed to be comprehensive, additional fees/costs may exist. *Access to UTP data and NASDAQ direct feed data may granted freely to academic institutions, see UTP Feed Pricing and NASDAQ Academic Waiver Policy for more info. **Historical data purchased from NYSE only covers 5/21/2018—present for NYSE National, thus an alternative data provider is required in ord [file pone.0226968.s006.pdf]

Data Provider	Feed(s)	One-time Cost	Monthly Fee
CTA	CQS, CTS		$1,850
UTP	UQDF, UTDF		*$2,500
NYSE	Integrated		$7,500
	Historical	$60,000	
NYSE ARCA	Integrated		$3,000
	Historical	$36,000	
NYSE MKT	Integrated		$2,500
(Now NYSE American)	Historical	$18,000	
National Stock Exchange NSX	Integrated		$0
(Now NYSE National)	Historical		*$1,500
NASDAQ	TotalView-ITCH		*$25,000
	Historical		*$1,250
NASDAQ BX	TotalView-ITCH		*$20,000
	Historical		*$500
NASDAQ PSX	TotalView-ITCH		*$17,000
	Historical		*$500
BATS BZX	Depth		$2,000
	Historical	$8,500	
BATS BYX	Depth		$2,000
	Historical	$8,500	
Direct Edge EDGA	Depth		$1,000
	Historical	$8,500	
Direct Edge EDGX	Depth		$2,000
	Historical	$8,500	
The Investors Exchange	TOPS	$0	$0
	DEEP	$0	$0
	Historical	$0	$0
Chicago Stock Exchange	CHX Book	$0	$0
(Now NYSE Chicago)	Historical		
Total		$148,000	$91,100

S2 Table. A Survey of Data Fees. The pricing presented in this table assumes a single consumer with an academic use case aiming to construct a dataset similar to what was used in this analysis. It is also assumes that non-display fees do not apply. Historical data costs assume a 12 month period of interest, i.e. calendar year 2016. Strictly speaking, historical data may sufficient for replicating the analysis presented in this paper, making subscription to live feeds unnecessary. However, utilizing historical data provided by each exchange excludes the possibility of collecting data from a single point of observation, reintroducing the issues of clock synchronization and relativity. Additionally, highlighting the monthly cost for comprehensive direct feed access shines a light on one of the reasons for the lack of academic participation in the analysis of modern U.S. stock markets. This does not include costs which may be incurred while curating the data, fulfilling potential co-location requirements, ISP/networking costs, computing hardware acquisition and maintenance, etc. DoB indicates that a product contains full Depth of Book information (adds, mod, and cancel messages), while ToB indicates that a product contains only Top of Book information (trade and quote messages). The NYSE Historical ToB product, also called NYSE Daily TAQ, is frequently used in academic studies due to it's relatively low cost and broad coverage (e.g. [**?**] use this product). Historical data from CHX is not directly available, and the live feeds are transitioning to NYSE technology, thus historical CHX data must be purchased from a third party. This list is not guaranteed to be comprehensive, additional fees/costs may exist. *Access to UTP data and NASDAQ direct feed data may granted freely to academic institutions, see UTP Feed Pricing and NASDAQ Academic Waiver Policy for more info. **Historical data purchased from NYSE only covers 5/21/2018 - present for NYSE National, thus an alternative data provider is required in order to obtain historical data from 2016. The sources used to construct this table include CTA feed pricing, UTP feed pricing via the Data Policies document, NYSE feed pricing, NYSE historical data pricing, NASDAQ feed pricing, BATS/DirectEdge feed pricing, and CHX feed pricing
