# Supplementary material for: Fragmentation and inefficiencies in US equity markets: Evidence from the Dow 30
Source: PLoS One. 2020 Jan 22;15(1):e0226968. doi: 10.1371/journal.pone.0226968 (PMC6975550; doi:10.1371/journal.pone.0226968)
Supplement: S3 Table — Trades that occurred during a dislocation in AAPL on 2016-01-07 at approximately 9:48am, more than three minutes after the trading “guardrails” are enforced. The “Delta” column indicates the difference between the Thesys timestamp and the SIP publication timestamp (in microseconds). For trade 0, Thesys received the trade at 9:48:55.396951 and the SIP timestamp was 9:48:55.396696. The “Extra” column contains additional deltas related to the timestamps added in the 2015 SIP changes, see [24] for additional details. In particular, this column contains the difference (in microseconds) between the Thesys timestamp and the exchange timestamp. For trade 0, Thesys received the trade at 9:48:55.396951 and the exchange timestamp was 9:48:55.397602, an example of the timestamp inversion seen in [24], which is generally cause by clock synchronization issues. (PDF) [file pone.0226968.s007.pdf]

Index	Date and time	delta	symbol	size	price	exchange	extra
0	2016-01-07 09:48:55.396951	255	AAPL	100	99.11	1	-651
1	2016-01-07 09:48:55.396951	227	AAPL	100	99.12	1	-651
2	2016-01-07 09:48:55.396978	237	AAPL	100	99.12	1	-678
3	2016-01-07 09:48:55.396978	222	AAPL	100	99.12	1	-678
4	2016-01-07 09:48:55.396978	204	AAPL	100	99.13	2	-852
5	2016-01-07 09:48:55.396998	207	AAPL	100	99.13	2	-872
6	2016-01-07 09:48:55.396998	190	AAPL	100	99.13	2	-872
7	2016-01-07 09:48:55.397064	239	AAPL	100	99.13	2	-938
8	2016-01-07 09:48:55.397064	216	AAPL	100	99.12	1	-764
9	2016-01-07 09:48:55.397068	204	AAPL	50	99.13	2	-942
10	2016-01-07 09:48:55.397196	316	AAPL	200	99.13	2	-1070
11	2016-01-07 09:48:55.397196	296	AAPL	100	99.16	1	-1013
12	2016-01-07 09:48:55.397196	279	AAPL	100	99.13	2	-1070
13	2016-01-07 09:48:55.397196	262	AAPL	395	99.11	3	-1044
14	2016-01-07 09:48:55.397297	344	AAPL	100	99.13	1	-997
15	2016-01-07 09:48:55.397297	327	AAPL	100	99.16	4	-1114
16	2016-01-07 09:48:55.397297	309	AAPL	100	99.13	1	-997
17	2016-01-07 09:48:55.397297	292	AAPL	100	99.14	2	-1171
18	2016-01-07 09:48:55.397297	275	AAPL	100	99.13	1	-997
19	2016-01-07 09:48:55.397297	259	AAPL	100	99.12	3	-1145
20	2016-01-07 09:48:55.397361	306	AAPL	100	99.14	2	-1235
21	2016-01-07 09:48:55.397431	358	AAPL	100	99.13	3	-1279
22	2016-01-07 09:48:55.397431	317	AAPL	100	99.14	2	-1305
23	2016-01-07 09:48:55.397431	298	AAPL	100	99.13	1	-1131
24	2016-01-07 09:48:55.397431	268	AAPL	100	99.13	3	-1279
25	2016-01-07 09:48:55.397499	316	AAPL	50	99.13	1	-1199
26	2016-01-07 09:48:55.397499	299	AAPL	100	99.13	3	-1347
27	2016-01-07 09:48:55.397499	284	AAPL	100	99.13	1	-1199
28	2016-01-07 09:48:55.397504	272	AAPL	100	99.14	2	-1378
29	2016-01-07 09:48:55.397504	255	AAPL	100	99.13	1	-1204
30	2016-01-07 09:48:55.397565	299	AAPL	50	99.13	3	-1413
31	2016-01-07 09:48:55.397565	281	AAPL	100	99.14	1	-1265
32	2016-01-07 09:48:55.397565	266	AAPL	200	99.13	3	-1413
33	2016-01-07 09:48:55.397604	290	AAPL	100	99.14	1	-1304
34	2016-01-07 09:48:55.397604	276	AAPL	100	99.13	3	-1452
35	2016-01-07 09:48:55.397604	260	AAPL	100	99.14	1	-1304
36	2016-01-07 09:48:55.397685	325	AAPL	100	99.14	3	-1533
37	2016-01-07 09:48:55.397685	309	AAPL	100	99.14	3	-1533
38	2016-01-07 09:48:55.397685	293	AAPL	100	99.14	1	-1385
39	2016-01-07 09:48:55.397731	323	AAPL	100	99.14	3	-1579
40	2016-01-07 09:48:55.397731	309	AAPL	100	99.14	1	-1431
41	2016-01-07 09:48:55.397731	294	AAPL	100	99.14	3	-1579
42	2016-01-07 09:48:55.397731	279	AAPL	50	99.15	1	-1431
43	2016-01-07 09:48:55.397767	300	AAPL	100	99.14	3	-1615
44	2016-01-07 09:48:55.397767	285	AAPL	100	99.14	3	-1615

Continued on next page

Index	Date and time	delta	symbol	size	price	exchange	extra
45	2016-01-07 09:48:55.397767	269	AAPL	900	99.15	3	-1615
46	2016-01-07 09:48:55.397824	310	AAPL	100	99.15	1	-1524
47	2016-01-07 09:48:55.397824	294	AAPL	100	99.15	3	-1672
48	2016-01-07 09:48:55.397824	280	AAPL	100	99.14	2	-1698
49	2016-01-07 09:48:55.397824	266	AAPL	100	99.15	2	-1698
50	2016-01-07 09:48:55.397870	298	AAPL	100	99.15	2	-1744
51	2016-01-07 09:48:55.397870	282	AAPL	100	99.15	1	-1570
52	2016-01-07 09:48:55.397894	290	AAPL	100	99.15	3	-1742
53	2016-01-07 09:48:55.397894	275	AAPL	100	99.15	1	-1594
54	2016-01-07 09:48:55.397894	260	AAPL	50	99.15	3	-1742
55	2016-01-07 09:48:55.397973	323	AAPL	50	99.15	1	-1673
56	2016-01-07 09:48:55.397973	307	AAPL	100	99.15	3	-1821
57	2016-01-07 09:48:55.397973	293	AAPL	100	99.15	1	-1673
58	2016-01-07 09:48:55.397994	299	AAPL	50	99.15	2	-1868
59	2016-01-07 09:48:55.398058	346	AAPL	50	99.16	1	-1758
60	2016-01-07 09:48:55.398058	331	AAPL	200	99.15	3	-1906
61	2016-01-07 09:48:55.398058	313	AAPL	100	99.16	1	-1758
62	2016-01-07 09:48:55.398125	366	AAPL	100	99.15	2	-1999
63	2016-01-07 09:48:55.398128	354	AAPL	100	99.15	3	-1976
64	2016-01-07 09:48:55.398147	357	AAPL	100	99.14	1	-1422
65	2016-01-07 09:48:55.398147	342	AAPL	200	99.15	2	-2021
66	2016-01-07 09:48:55.398158	339	AAPL	100	99.16	3	-2006
67	2016-01-07 09:48:55.398177	342	AAPL	100	99.15	2	-2051
68	2016-01-07 09:48:55.398225	375	AAPL	100	99.16	3	-2073
69	2016-01-07 09:48:55.398225	359	AAPL	5	99.16	3	-2073
70	2016-01-07 09:48:55.398225	345	AAPL	100	99.14	1	-1500
71	2016-01-07 09:48:55.398267	373	AAPL	100	99.15	1	-1542
72	2016-01-07 09:48:55.398267	358	AAPL	100	99.16	1	-1542
73	2016-01-07 09:48:55.398267	342	AAPL	100	99.17	1	-1542
74	2016-01-07 09:48:55.398267	327	AAPL	100	99.17	1	-1542
75	2016-01-07 09:48:55.398267	312	AAPL	50	99.17	1	-1542
76	2016-01-07 09:48:55.398272	300	AAPL	400	99.11	5	-1967
77	2016-01-07 09:48:55.398272	285	AAPL	100	99.12	5	-1967
78	2016-01-07 09:48:55.398386	384	AAPL	100	99.13	5	-2081
79	2016-01-07 09:48:55.398414	397	AAPL	100	99.13	5	-2109
80	2016-01-07 09:48:55.398414	381	AAPL	100	99.13	5	-2109
81	2016-01-07 09:48:55.398414	365	AAPL	100	99.13	5	-2109
82	2016-01-07 09:48:55.398444	381	AAPL	50	99.13	5	-2139
83	2016-01-07 09:48:55.398444	366	AAPL	100	99.13	5	-2139
84	2016-01-07 09:48:55.398444	352	AAPL	100	99.13	5	-2139
85	2016-01-07 09:48:55.398444	337	AAPL	100	99.13	5	-2139
86	2016-01-07 09:48:55.398444	322	AAPL	100	99.14	5	-2139
87	2016-01-07 09:48:55.398532	395	AAPL	50	99.14	5	-2227
88	2016-01-07 09:48:55.398532	369	AAPL	100	99.15	2	-1507
89	2016-01-07 09:48:55.398532	354	AAPL	100	99.16	2	-1507

Continued on next page

Index	Date and time	delta	symbol	size	price	exchange	extra
90	2016-01-07 09:48:55.398537	344	AAPL	50	99.17	2	-1512
91	2016-01-07 09:48:55.398537	330	AAPL	100	99.17	2	-1512
92	2016-01-07 09:48:55.398560	339	AAPL	100	99.17	2	-1535
93	2016-01-07 09:48:55.398560	324	AAPL	50	99.17	3	-1282
94	2016-01-07 09:48:55.398560	309	AAPL	100	99.14	5	-1434
95	2016-01-07 09:48:55.398571	305	AAPL	50	99.15	5	-1445
96	2016-01-07 09:48:55.398571	291	AAPL	100	99.15	5	-1445

S3 Table. Example AAPL Trades. Trades that occurred during a dislocation in AAPL on 2016-01-07 at approximately 9:48am, more than three minutes after the trading "guardrails" are enforced. The "Delta" column indicates the difference between the Thesys timestamp and the SIP publication timestamp (in microseconds). For trade 0, Thesys received the trade at 9:48:55.396951 and the SIP timestamp was 9:48:55.396696. The "Extra" column contains additional deltas related to the timestamps added in the 2015 SIP changes, see [1] for additional details. In particular, this column contains the difference (in microseconds) between the Thesys timestamp and the exchange timestamp. For trade 0, Thesys received the trade at 9:48:55.396951 and the exchange timestamp was 9:48:55.397602, an example of the timestamp inversion seen in [1], which is generally cause by clock synchronization issues.

References

1. Bartlett RP, McCrary J. How rigged are stock markets? Evidence from microsecond timestamps. Journal of Financial Markets. 2019;.
