# Supplementary material for: Fragmentation and inefficiencies in US equity markets: Evidence from the Dow 30
Source: PLoS One. 2020 Jan 22;15(1):e0226968. doi: 10.1371/journal.pone.0226968 (PMC6975550; doi:10.1371/journal.pone.0226968)
Supplement: S4 Table — A subset of the trades from S3 Table that resulted in positive ROC. Positive ROC indicates that these trades received favorable prices that were aligned with the SIP NBBO. (PDF) [file pone.0226968.s008.pdf]

timestamp	exchange	price	shares	direct_bid	direct_ask	sip_bid	sip_ask	roc
2016-01-07 09:48:55.396951	1	99.11	100	99.14	99.14	99.10	99.11	3.0
2016-01-07 09:48:55.397196	3	99.11	395	99.14	99.15	99.10	99.11	15.8
2016-01-07 09:48:55.398147	1	99.14	100	99.16	99.17	99.12	99.14	3.0
2016-01-07 09:48:55.398225	3	99.14	100	99.16	99.17	99.12	99.14	3.0
2016-01-07 09:48:55.398532	2	99.15	100	99.16	99.17	99.14	99.15	2.0
2016-01-07 09:48:55.398560	5	99.14	100	99.16	99.17	99.14	99.14	3.0

S4 Table. Example AAPL Trades with Positive ROC. A subset of the trades that occurred during a dislocation in AAPL on 2016-01-07 at approximately 9:48am that resulted in positive ROC. Positive ROC indicates that these trades received favorable prices that were aligned with the SIP NBBO.
