# Supplementary material for: Fragmentation and inefficiencies in US equity markets: Evidence from the Dow 30
Source: PLoS One. 2020 Jan 22;15(1):e0226968. doi: 10.1371/journal.pone.0226968 (PMC6975550; doi:10.1371/journal.pone.0226968)
Supplement: S5 Table — A subset of the trades from S3 Table that resulted in negative ROC. Negative ROC indicates that these trades executed at less favorable prices than what was offered by the DBBO. (PDF) [file pone.0226968.s009.pdf]

timestamp	exchange	price	shares	direct_bid	direct_ask	sip_bid	sip_ask	roc
2016-01-07 09:48:55.398272	5	99.12	100	99.16	99.17	99.12	99.14	-4
2016-01-07 09:48:55.398386	5	99.13	100	99.16	99.17	99.13	99.15	-3
2016-01-07 09:48:55.398444	5	99.14	100	99.16	99.17	99.14	99.15	-2
2016-01-07 09:48:55.398532	5	99.14	50	99.16	99.17	99.14	99.15	-1
2016-01-07 09:48:55.398560	5	99.14	100	99.16	99.17	99.14	99.14	-2

S5 Table. Example AAPL Trades with Negative ROC. A subset of the trades that occurred during a dislocation in AAPL on 2016-01-07 at approximately 9:48am that resulted in negative ROC. Negative ROC indicates that these trades executed at less favorable prices than what was offered by the DBBO.
